# K Value: An Indicator that can Characterize the Cold and Hot Properties of Traditional Chinese Medicines

**DOI:** 10.3389/fphar.2022.877102

**Published:** 2022-05-12

**Authors:** Meina Yang, Jingxiang Pang, Zhongwen Zhang, Jialei Fu, Hua Fan, Yufeng Zhang, Lingyuan Min, Baochen Zhou, Jinxiang Han

**Affiliations:** ^1^ Department of Clinical Pharmacy, The First Affiliated Hospital of Shandong First Medical University, Shandong Medicine and Health Key Laboratory of Clinical Pharmacy, Jinan, China; ^2^ Key Laboratory of Biotechnology Drug (Shandong Academy of Medical Sciences), Biomedical Sciences College and Shandong Medicinal Biotechnology Centre, Shandong First Medical University and Shandong Academy of Medical Sciences, Jinan, China; ^3^ Department of Endocrinology, The First Affiliated Hospital of Shandong First Medical University, Jinan, China; ^4^ Shandong Academy of Chinese Medicine, Jinan, China

**Keywords:** Chinese herbs, k value, cold and hot property, delayed luminescence, scenedesmus obliquus

## Abstract

**Aims:** The cold and hot properties of Chinese medicines are an important concept to represent the function of drugs, and are also a unique classification method of traditional Chinese medicine (TCM). The method reflects an herb’s therapeutic properties and guides reasonable clinical prescription. However, the present key problem is the lack of an objective and quantitative evaluation index for the cold and hot properties of Chinese herbs. Delayed luminescence (DL) is the long-term afterglow of biological systems after illumination with light, which can reflect differences in herbal materials prepared under different conditions. We aim to use S. obliquus as an indicator organism to characterize the differences between the cold and hot properties of Chinese herbs.

**Methods:** Scenedesmus obliquus (S. obliquus) was used as an indicator organism to characterize the differences between the cold and hot properties of Chinese herbs. The decoction solution of different properties of Chinese herbs was added to S. obliquus culture medium; then, the delayed luminescence (DL) of S. obliquus after the addition of decoctions of different properties of Chinese herbs was measured to obtain information on the effect of different properties of Chinese herbs on S. obliquus. Many DL parameters were calculated, and ROC curve analysis was applied with the aim of finding a suitable parameter that can characterize the differences in cold and hot properties of Chinese herbs.

**Results:** Our results show that the K value is a sensitive parameter that can reflect the differences of cold and hot properties of Chinese herbs, thus providing new insights into the cold and hot properties of Chinese herbs.

**Conclusions:** DL measurement of S. obliquus after addition of different properties of Chinese herbs could be a novel and promising method to study the cold and hot properties of Chinese herbs.

## Introduction

The cold and hot properties of Chinese medicines are an important concept to represent the function of drugs, which reflects the action tendency of drugs. Cold medicine is mainly used to inhibit the body’s functional activities, and hot medicine is mainly used to stimulate the body’s functional activities. However, as the theoretical basis of clinical medicine, the traditional Chinese medicine (TCM) medicinal property theory utilizes philosophy rather than the scientific method as its theoretical tool. Its theory abstracting, concept blurring, and diagnosis and examination method subjectively lacks the rigor and evidence of modern science. Therefore, there is an urgent need to obtain an objective and quantitative evaluation indicator of TCM medicinal properties.

At present, there are two problems in modern research on the cold and hot properties theory of Chinese medicine. Firstly, most studies are still based on indirect measurement or correlation analysis and have not carried out research based on the objective fact that Chinese medicine regulates the syndrome of cold and hot at the macro level, which defines the cold and hot properties of Chinese medicine (Liang et al., 2013). A holistic view is the most important characteristic of TCM. The other is the lack of appropriate detection methods, which can reflect the cold and hot properties of medicinal herbs from a macro and whole-body view by observing the impacts of drugs (Zhang et al., 2009).

Delayed luminescence (DL) is the long-term afterglow of biological systems after illumination with white light. There is a close connection between the biological state liquid inorganic systems of the system itself and the parameters of DL (Grasso et al., 2016; Brizhik et al., 2001). DL has been used as a tool for directly and rapidly assessing biological systems and has been found to provide a sensitive indicator of food quality (Scordino et al., 2008; Egerer et al., 2008). In addition, delayed luminescence detection has been applied in tumor cell discrimination, disease diagnosis, and especially TCM quality assessment (Kim et al., 2005; Ping et al., 2012; Pang et al., 2016; Pang et al., 2016; Sun et al., 2016). Recent studies examined the DL properties that can be used to indicate differences in herbal materials prepared under different conditions, including the processing method and growing environment (Sun et al., 2016; Sun et al., 2017). These differences in DL properties reflect variations in the therapeutic properties of Chinese herbs. DL may provide an integrated, comprehensive view of the nature and property of Chinese herbs. A number of studies have shown that delay luminescence (DL) can be expressed as (Gu et al., 2012; Popp et al., 2002; Sun M. et al., 2016):

$$I\left(t\right)=A\csc h^{2}\left(\frac{t}{B}+c\right),$$ (1)

$$I_{W}=\frac{AB}{W}\left[\coth C-\coth\left(\frac{W}{B}+C\right)\right],$$ (2)

$$I_{W}=Kt+b\;,$$ (3)

$$I\left(t\right)=\frac{I_{0}}{{\left(1+t/\tau \right)}^{\beta }},$$ (4)

$$I\left(t\right)=y_{0}+A_{1}e^{-t/t_{1}}+A_{2}e^{-t/t_{2}}.$$ (5)

With these models, the delay luminescence data can be well fitted. Equations 1–3 are called Gu function (Gu et al., 2012), in the Eq. 1, A is an intensity parameter, which depends on the properties of the sample under test and is also related to the system structure and lighting conditions; B is a characteristic time, which is only related to the properties of the sample itself; C is a phase factor, which can sensitively determine the initial intensity of luminescence, regardless of the system structure and lighting conditions. DL characteristics are described by A, B and C, which can be obtained by fitting the experimental data. Equation 2 is the calculation formula of average intensity, W is the total measurement time, the average intensity (I_W_) of seven times of excitation delayed luminescence is linearly fitted with the measurement time t. The slope (K) of the obtained linear fitting Eq. 3 represents the dynamic evolution behavior of delayed luminescence in different liquid environments of S. obliquus, and it is a reliable parameter to characterize the properties of samples. The Eq. 4 is a hyperbolic function formula, I_0_ is the initial intensity of the delayed luminescence after excitation, and β is an index factor associated with the rate of decay (Popp et al., 2002). The Eq. 5 is two-exponential decay function, where A_1_ and A_2_ are the amplitudes of photon emission of exponential decay components, t_1_ and t_2_ are time constants for the exponential decays, and y_0_ represents the final value of photon emission in the DL decay curve (Sun MM et al., 2016).

Algae in water bodies are very sensitive to toxic pollutants. They can live in different types of water bodies and different habitats of water bodies, and they have therefore been used as indicator organisms for monitoring water quality changes. S. obliquus is a common planktonic alga in freshwater, easy to culture artificially, and very sensitive to its environment, and its biological behavior is different in different aquatic environments (Dewez et al., 2008; Xin et al., 2014). Therefore, the species is often used as a good experimental material for studying water pollution. Previous studies demonstrated that the luminescence emission behaviors of different chemical compositions on S. obliquus were very different, which were applied to the evaluation of aquatic environments and food pollution (Han et al., 2016; Katsumata et al., 2017). This finding provides a clue that S. obliquus could also be a promising indicator for characterizing different properties of Chinese herbs.

Here, we aim to use S. obliquus as an indicator organism to characterize the differences between the cold and hot properties of Chinese herbs. The decoction solution of different properties of Chinese herbs was added to S. obliquus culture medium; then, the delayed luminescence (DL) of S. obliquus after the addition of decoctions of different properties of Chinese herbs was measured to obtain information on the effect of different properties of Chinese herbs on S. obliquus. Many DL parameters were calculated, and ROC curve analysis was applied with the aim of finding a suitable parameter that can characterize the differences in cold and hot properties of Chinese herbs.

## Materials and Methods

### Herbs Prepared

According to the Chinese Pharmacopoeia (V.2015), a total of 160 herbs were selected, including 74 cold herbs and 86 hot herbs. In addition, a pair of Chinese herbs with medicine property changes before and after processing were also collected (Arisaematis Rhizoma Preparatum and Arisaema cum Bile). Arisaematis Rhizoma Preparatum is a hot herb, and Arisaema cum Bile is a cold herb after being processed by Arisaematis Rhizoma Preparatum. All herbs were bought from the Jinan Jianlian Chinese medicinal herb store and identified by Prof. Yuanbin Zhang of Shandong Academy of Medical Sciences.

Every herb was weighed (10 g) before being decocted twice, namely, the first boiling and second boiling. The first boiling takes place as follows: 200 ml water is added to 10 g Chinese herb, the mixture is soaked in a dish for half an hour, the electric stove is switched on, the power is to 2000 W, decoction is performed until boiling, the power is adjusted to 800 W, and decoction occurs for 15 min. After the decoction is finished, the liquid is filtered with absorbent cotton gauze, and the residue is filtered twice. The second boiling takes place as follows: 200 ml water is added to the head residue, decoction is performed again for 20 min, the residue is filtered out, and the liquid is preserved. The two decoctions were combined and filtered with filter paper. The final decoction was 50 ml. The decoction was cooled to room temperature and stored in a 50 ml sterile centrifuge tube. The resulting extracts were decanted, filtered and evaporated to 0.2 g/ml.

### Scenedesmus Obliquus

S.obliquus was purchased from the Institute of Hydrobiology, Chinese Academy of Sciences (No. FACHB-933). S obliquus was grown in BG11 medium (Institute of Hydrobiology, Chinese Academy of Sciences, China) at 25°C in a light incubator (Jiangnan, GZX, China). Light intensity was 4,000 lux, sunshine duration was 12 h, and the bottle was shaken twice a day.

Two months after the culture, the transfer was performed, and the sterile operation was performed. The transfer ratio was 1:3, that is, 300 ml BG11 culture medium was added to 100 ml algal solution, and the transfer was continued in the light culture box. S. obliquus was used as an indicator organism to study the effect of different kinds of Chinese herb decoctions.

### Delayed Luminescence Measurements

Before the measurement, the concentration of S. obliquus was adjusted to 3 × 10^7^ counts/ml. In total, 3 ml of well-grown S. obliquus was placed in a quartz colorimetric dish (4 × 1 × 1 cm). The decoction of Chinese herb was added to the S. obliquus solution in a proportion of 1:10. Mixed liquors were placed in the chamber of a photomultiplier system. The photomultiplier system was designed for measurement of DL of S. obliquus. The device is equipped with a black chamber with a single opening for a cooled single-photon-count photomultiplier tube (QA9863; ET Enterprises, United Kingdom). A shutter was placed between the photomultiplier tube and the chamber. The chamber contains a sample holder suitable for a 10 cm petri dish. For excitation, computer-regulated LEDs (70 mm, 10 W, type LZ4-00MD00; LED Engine Inc., United States) were used. The excitation was carried out with an LED (LED Engine Inc., white light). After dark adaptation for 15 min, the sample was excited by light for 10 s; then, recording was performed with consecutive 900 s periods, with an internal time of 1 s. Then, after dark adaptation for 15 min, the second measurement was performed. After six repeat measurements, seven DL curves were obtained that carried the information of the effect of different properties of Chinese herbs on S. obliquus with time. The total measurement for every Chinese herb was 210 min.

### Statistical Analysis

Statistical analysis was performed using Statistica 10.0 software (StatSoft, United States). Nonlinear fitting analyses were carried out to obtain the delay luminescence kinetic parameters. ROC curve analyses were carried out (SPSS 19.0 software) to obtain the sensitivity, specificity and threshold of discrimination of cold and hot properties of Chinese herbs. Spearman’s rank correlation (ρ) was used to quantify the correlation between the bioactive constituents and DL parameters (SPSS version 19.0). Moderately strong significant correlations were defined as Spearman’s (ρ) > 0.35 and *p* < 0.01, respectively. For the comparisons between the data from two different properties of samples, the differences of parameters were tested with the independent-sample test (SPSS version 19.0). *p* values of less than 0.05 were considered significant.

## Result

### Delayed Luminescence (DL) Curves for Typical Hot and Cold Chinese Herbs

**FIGURE 1:**
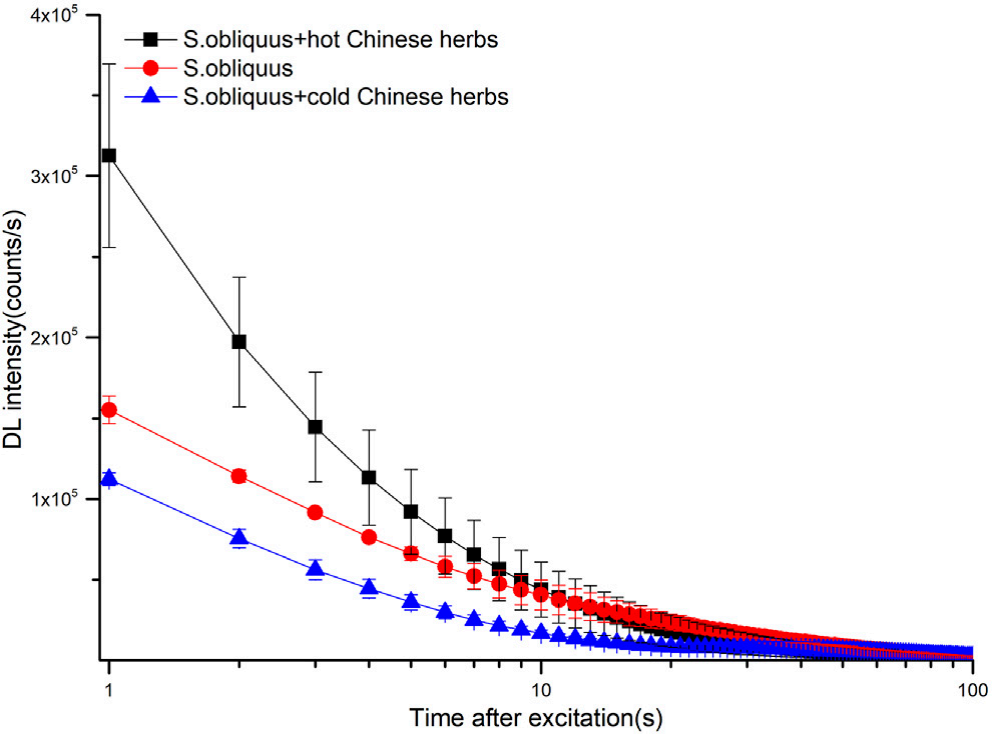
DL decay curves for S. obliquus, S. obliquus +hot Chinese herbs and S. obliquus + cold Chinese herbs. Data are plotted as the mean ± SEM. Note that the data are plotted on a log-linear scale.

S.obliquus is very sensitive to its environment, and its biological behavior will be different in different living environments. The DL behaviors of different chemical compositions on S. obliquus were very different (Baky et al., 2014). Therefore, take three typical cold and three typical hot Chinese herbs for example, the DL after addition of their decoction were measured, and the DL decay curves for them were shown in Figure 1. The hot and cold Chinese herbs decoction showed significantly different DL curves compared with the S. obliquus itself,and the initial intensity of the hot Chinese herb is significantly higher than the cold Chinese. Hot Chinese herbs decoction increased the intensity of DL, but cold Chinese herb decoction weakened the intensity of DL, especially in the first 10 s after excitation. Next, we measured the DL profiles of 160 Chinese herbal samples (including 74 cold and 86 hot samples) in order to ensure the difference between cold and hot properties of Chinese herbs. 160 Chinese herbs were analyzed, their English and Latin names and their properties (cold or hot) in accordance with the 2015 Chinese Pharmacopoeia were listed as Supplementary Material.

**FIGURE 2:**
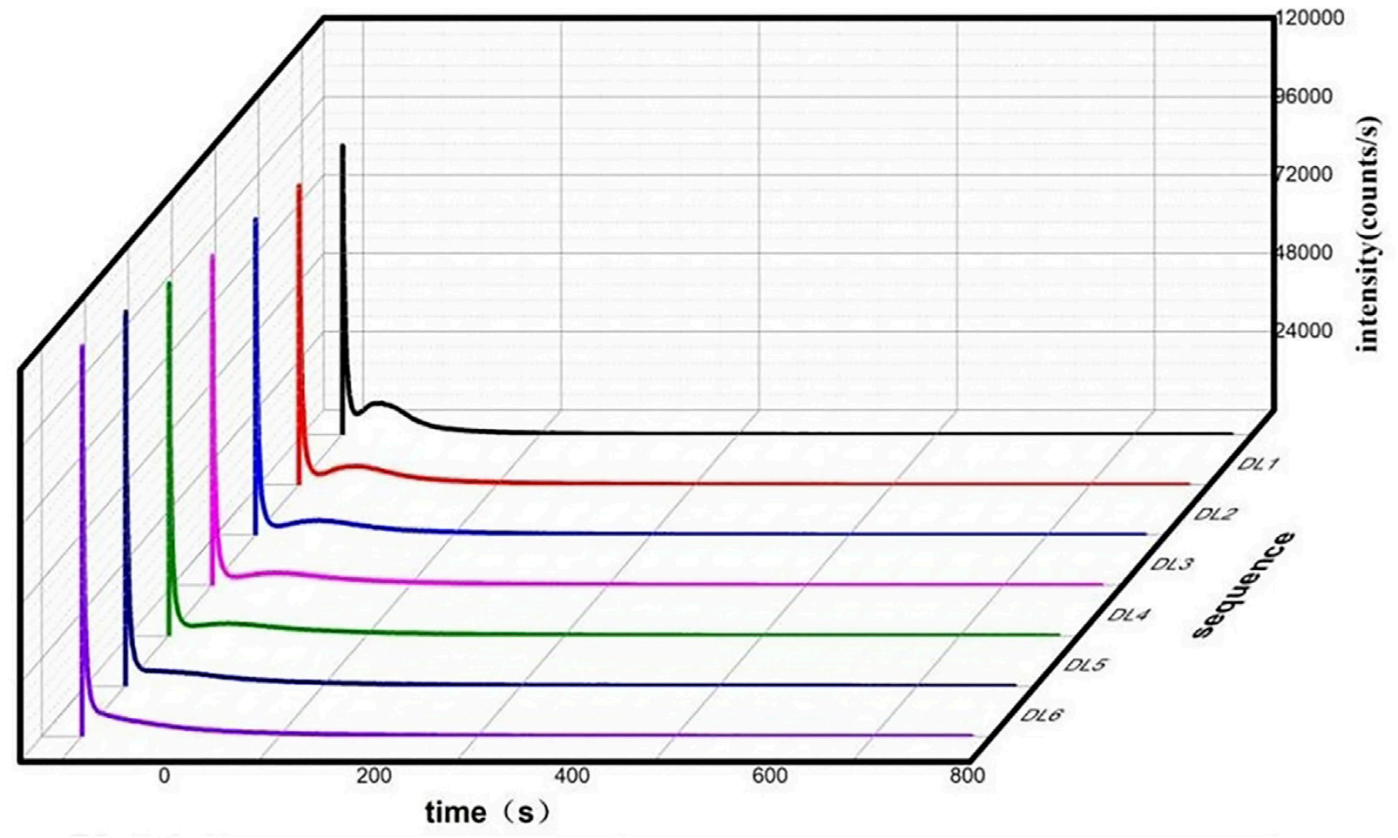
Seven DL decay curves for S. obliquus with time.

Every herbal sample was measured seven times according to the same procedure described in the Materials and Methods, with the aim to obtain the dynamic behavior of the DL of S. obliquus after addition of herbal samples with the time. The seven DL decay curves of S. obliquus are shown in Figure 2. The result shows that the intensity of DL increases gradually after multiple excitations.

### The Differences of DL Parameters Between the Hot and Cold Chinese Herbs

**FIGURE 3:**
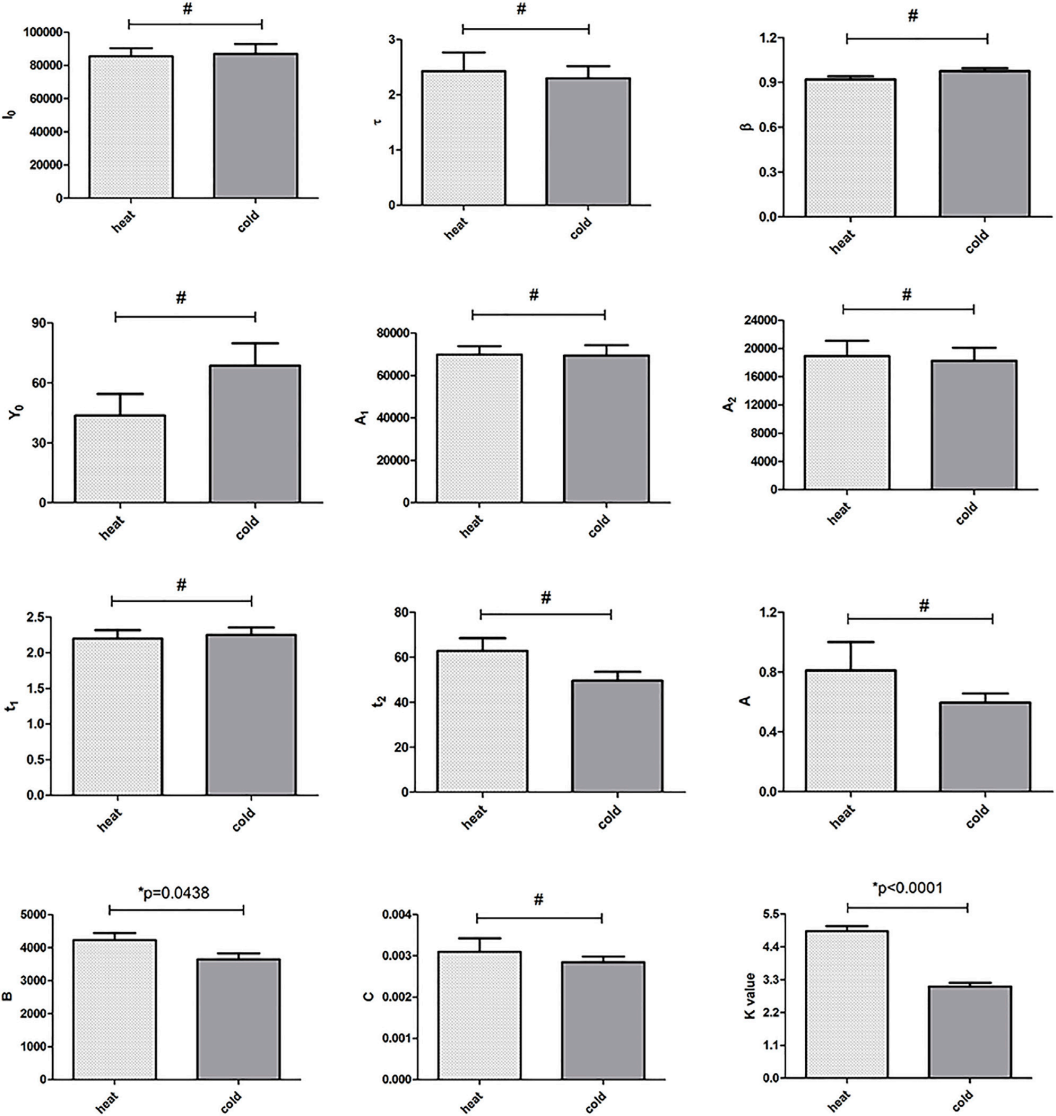
Histograms comparing the DL parameters obtained between the two different properties of Chinese herb groups (hot and cold). #*p* > 0.05, *p < 0.05 (two-tailed unpaired Student’s t-test).

On average, the DL parameters in the hot group were larger than those in the cold group (Figure 3). To analyze the difference between DL parameters, a two-tailed unpaired Student’s t-test was used to compare the 12 DL parameters between the hot and cold groups. The analysis revealed that only two parameters differed significantly between the two groups, namely, B of the Gu function and the slope of linear fitting of 7 I_W_ (named the K value) (*p* = 0.00438 and *p* < 0.0001, respectively); the 10 other parameters did not differ significantly. The result also shows that the K value could be a more appropriate parameter that can reflect the differences in cold and hot properties of Chinese herbs.

### ROC Curve Analysis for the DL Parameter K Value

**FIGURE 4:**
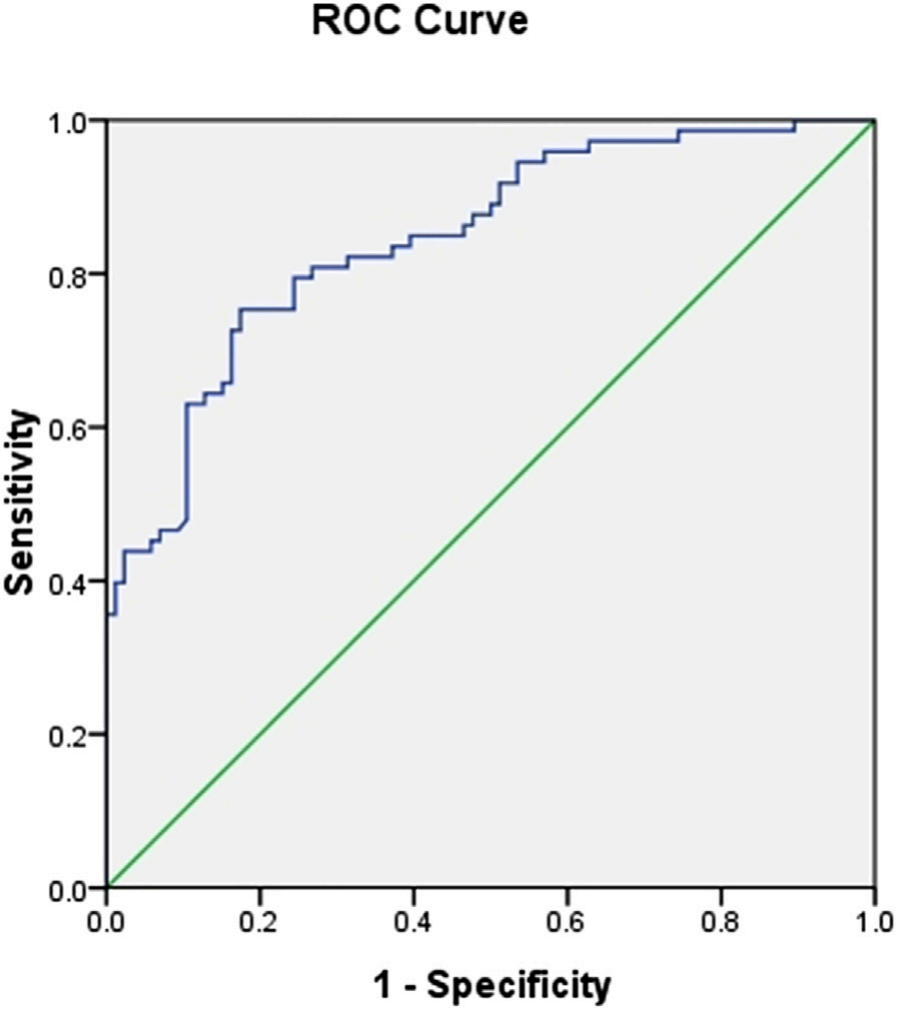
Receiver operating characteristic (ROC) curve analysis for the DL parameter K value. The AUC_k value_ is 0.844.

To assess the effect of the parameter K value on the ability to discriminate hot and cold Chinese herbs, receiver operating characteristic (ROC) analysis was applied to determine optimal sensitivity and specificity to discriminate between the cold and hot properties of Chinese herbs. Figure 4 displays the ROC analysis result. The result shows that the area under ROC curve (AUC) was 0.844 (95% CI, 0.784–0.904). The K value at an RT > 3.8526 (sensitivity = 0.808, specificity = 0.733) maximized separation of hot Chinese herbs from cold Chinese herbs.

### K Value Could Reflect the Property Change of Chinese Herbs

**FIGURE 5:**
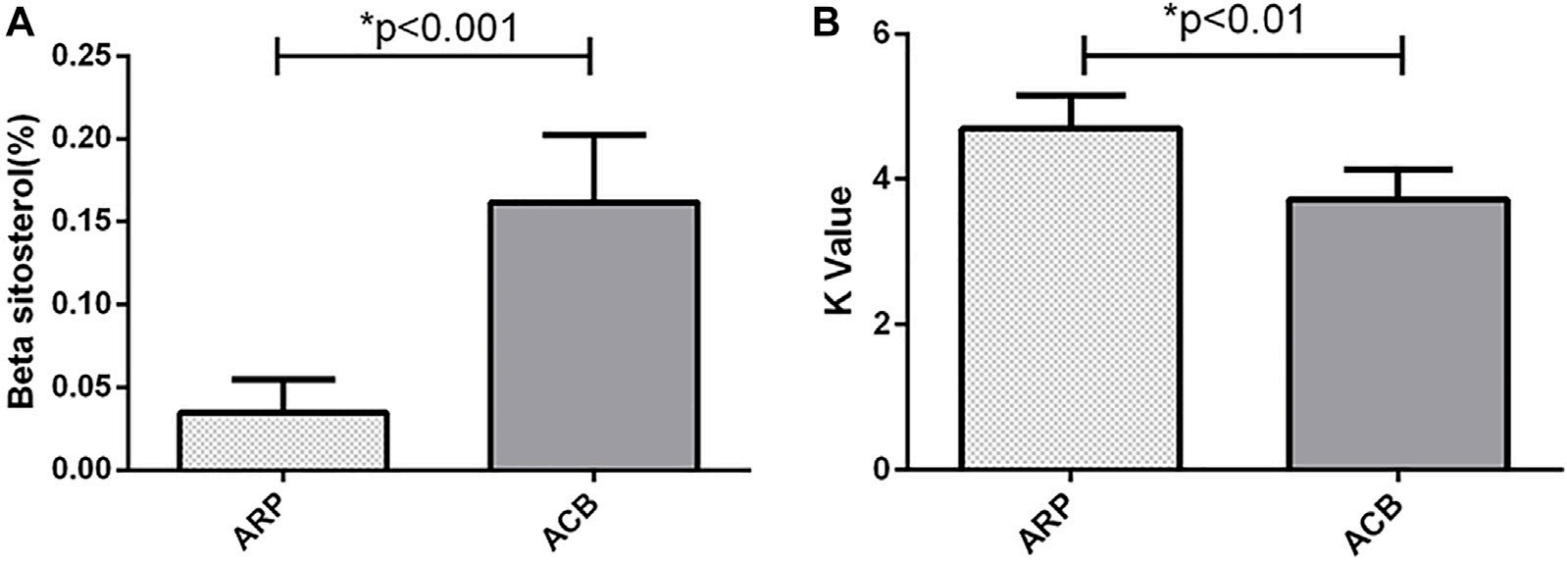
**(A)** The content of β-sitosterol of Arisaematis Rhizoma Preparatum (ARP) and Arisaema cum Bile (ACB) (*p < 0.001). **(B)** DL parameter K value of the decoction of Arisaematis Rhizoma Preparatum and Arisaema cum Bile (*p < 0.01).

To support our data that the DL parameter K value may reflect the property change of Chinese herbs, we selected a pair of Chinese herbs for which the medicine property changes before and after processing (i.e., Arisaematis Rhizoma Preparatum and Arisaema cum Bile). Arisaematis Rhizoma Preparatum is a hot herb, and Arisaema cum Bile is a cold herb after being processed by Arisaematis Rhizoma Preparatum. The DL parameter K value was analyzed before and after the processing of Arisaematis Rhizoma Preparatum (Figure 5A), and their main bioactive constituent (beta-sitosterol) was also detected using HPLC (Figure 5B). Figure 5A illustrates the difference in β-sitosterol before and after processing of ARP by HPLC. The result shows that the content of β-sitosterol in ARP is significantly lower than that in ACB (*p* = 0.0031). Figure 5B illustrates the difference in the DL parameter K value before and after processing of ARP. The result shows that the K value of ARP is significantly higher than that of ACB (*p* = 0.00014). Both the chemical analysis and the DL measurements successfully identified differences in ARP and ACB based on the cold and hot properties of these Chinese herbs. Then, we determined the correlations between the chemical constituents and DL parameter K value using Spearman’s correlation method. The results demonstrate that there is a significant negative correlation between the K value and β-sitosterol (*r* = −0.603, *p* = 0.0026).

## Discussion

The scientific connotation and quantification of the cold and hot properties of Chinese herbs are the key issues associated with the modernization and internationalization of TCM. The cold and hot properties of Chinese herbs are formed by the accumulation of long-term experience and have been an effective guide to the clinical practice of TCM. However, due to the differences in history, culture, region, academic school, academic level, clinical experience, *etc*., scholars of ancient and modern times often have different descriptions of the cold and hot medicinal properties of the same drug, some of which are even the opposite of each other. This phenomenon not only causes difficulties in learning and confusion in clinical application but also hinders the academic development of TCM and seriously affects modern research (Han et al., 2011; Pang et al., 2012).

Since the 1970s, scientists from China, Japan, and Korea have been applying modern scientific methods, including molecular, optical, physiological and pharmacological techniques, to explore the material basis or physiological and metabolic changes related to the cold and hot properties of Chinese herbs [21] (Qin et al., 2005). However, all these results have not been widely accepted. The reason lies in the absence of a holistic view that conforms to the development law of TCM theory, and the absence of a scientific research method suitable for modern research on TCM theory. As a result, new technologies are constantly being adopted. Some scientists analyzed the impacts of herbs on animal behaviors and functions, aiming to establish an objective evaluation method for determining the cold and hot properties of herbal medicines (Zhang et al., 2009; Zhao et al., 2011). Because of the complexity of experimental animals, although the approach of animal thermotropic behavior is a holistic approach, it is time consuming, strenuous and inaccurate because of the nonconformity of the animal.

Studies have reported that S. obliquus is one of the most widely used species in bioassays of water environments; the change in ultra-weak emissions caused by the cells’ metabolism and density has been the most valid and common indicator (Mur et al., 1974; Li et al., 2011). This indicator has an advantage, since it represents an integrating parameter showing water contamination. This is consistent with the holistic view of TCM. In previous studies, the DL of S. obliquus showed very good repeatability and stability. After adding decoctions of different properties of Chinese medicines, the DL of S. obliquus obviously changed, as shown in Figure 1. These results suggest that the DL of S. obliquus with cold and hot properties of Chinese herbs can be used to reflect the nature and function of Chinese herbs.

In this study, we detected the DL of S. obliquus after addition of 160 kinds of cold and hot properties of Chinese herbs. The DL curves of 160 Chinese herbs were detected using a photomultiplier system and analyzed based on many fitting formulas as described above. Many DL parameters were obtained. According to the statistical analysis of these many DL parameters, we found a promising DL parameter (i.e., the slope (K value) of the linear fitting equation of 7 I_W_), which could reflect the differences between the cold and hot properties of Chinese herbs. The K value represents the dynamic change in the behavior of delayed luminescence of S. obliquus in different liquid environments, and it is a relatively reliable parameter for characterizing the properties of herb samples. The significant differences in K values between cold and hot properties of Chinese herbs suggests that the K value may be used as a promising indicator of the cold and hot properties of Chinese medicine. To assess the effect of the DL parameter K value on the ability to discriminate hot and cold properties of Chinese herbs, a receiver operating characteristic (ROC) analysis was applied.

To support our finding that the DL parameter K value may reflect the medicine property change of Chinese herbs, we selected a pair of Chinese herbs that had medicine property changes before and after processing (i.e., ARP and ACB). The hot property of ARP can be changed into a cold property (i.e., ACB) after being processed. The effective components and K value of the two herbs were analyzed. The K value of ACB (cold) was lower after processing by ARP (hot). The main chemical component (β-sitosterol) of ARP and ACB was detected, and the results showed that β-sitosterol in ACB (cold) was higher after processing by ARP (hot). Then, Spearman’s correlation analysis between the β-sitosterol and K value was applied, and the results demonstrated that the K value was significantly negatively correlated with β-sitosterol. β-sitosterol is a main contributor to the cold property of ACB(Moon et al., 2007; Park et al., 2007).

K value of ACB is lower than that of ARP may be due to the effect of β-sitosterol. The result further confirmed that the DL parameter K value of S. obliquus after the addition of different Chinese herbs could reflect the difference between different properties of Chinese herbs. DL measurement of S. obliquus after addition of different properties of Chinese herbs could be a novel and promising method to study the cold and hot properties of Chinese herbs.

## Conclusion

The nature of the properties of Chinese herbs is the key issue associated with the modernization and internationalization of TCM. There is an urgent need to obtain an objective and quantitative method to characterize the different properties of Chinese herbs. In this paper, we introduced a novel method to characterize such differences (i.e., the use of S. obliquus as an indicator organism to characterize the differences between cold and hot properties of Chinese herbs). A decoction solution of different properties of Chinese herbs was added to a solution containing S. obliquus; then, the delayed luminescence (DL) of S. obliquus after the addition of different properties of Chinese herbs was measured to obtain information on the effect of different properties of Chinese herbs on S. obliquus. Our results show that the K value is a sensitive parameter that can reflect the differences in the cold and hot properties of Chinese herbs. DL measurement of S. obliquus after the addition of different properties of Chinese herbs could be a novel and promising method to study the cold and hot properties of Chinese herbs.

## Data Availability

The original contributions presented in the study are included in the article/Supplementary Material, further inquiries can be directed to the corresponding author.
